# Addition of Adipose-Derived Stem Cells to Mesenchymal Stem Cell Sheets Improves Bone Formation at an Ectopic Site

**DOI:** 10.3390/ijms17020070

**Published:** 2016-02-02

**Authors:** Zhifa Wang, Zhijin Li, Taiqiang Dai, Chunlin Zong, Yanpu Liu, Bin Liu

**Affiliations:** 1 State Key Laboratory of Military Stomatology, Department of Oral and Maxillofacial Surgery, School of Stomatology, Fourth Military Medical University, Xi’an 710032, China; wangzf19871013@163.com (Z.W.); lizhijin@fmmu.edu.cn (Z.L.); daitaiqiang5168@163.com (T.D.); mujiezi23@163.com (C.Z.); 2 State Key Laboratory of Military Stomatology, Department of Oral Biology, School of Stomatology, Fourth Military Medical University, Xi’an 710032, China

**Keywords:** mesenchymal stem cells, cell sheets, adipose-derived stem cells, bone regeneration

## Abstract

To determine the effect of adipose-derived stem cells (ADSCs) added to bone marrow-derived mesenchymal stem cell (MSC) sheets on bone formation at an ectopic site. We isolated MSCs and ADSCs from the same rabbits. We then prepared MSC sheets for implantation with or without ADSCs subcutaneously in the backs of severe combined immunodeficiency (SCID) mice. We assessed bone formation at eight weeks after implantation by micro-computed tomography and histological analysis. In osteogenic medium, MSCs grew to form multilayer sheets containing many calcium nodules. MSC sheets without ADSCs formed bone-like tissue; although neo-bone and cartilage-like tissues were sparse and unevenly distributed by eight weeks after implantation. In comparison, MSC sheets with ADSCs promoted better bone regeneration as evidenced by the greater density of bone, increased mineral deposition, obvious formation of blood vessels, large number of interconnected ossified trabeculae and woven bone structures, and greater bone volume/total volume within the composite constructs. Our results indicate that although sheets of only MSCs have the potential to form tissue engineered bone at an ectopic site, the addition of ADSCs can significantly increase the osteogenic potential of MSC sheets. Thus, the combination of MSC sheets with ADSCs may be regarded as a promising therapeutic strategy to stimulate bone regeneration.

## 1. Introduction

The field of bone tissue engineering seeks to promote the repair of bone defects caused by congenital deformities, trauma, and tumor resection [1]. Among the many challenges to generating tissue-engineered bone, angiogenesis remains the most critical issue to be addressed because a good vascular supply is necessary for transport of nutrients and metabolic waste [2]. In recent years, co-implantation or co-culture systems have been widely studied, and some promising results have been reported, showing that such systems can significantly improve bone formation [3]. For example, co-implantation of mesenchymal stem cell (MSC) sheets with endothelial progenitor cells (EPCs) differentiated from MSCs was shown to not only increase bone formation but also to generate a vascular network [4]. Co-cultures of peripheral blood CD34+ cells and MSCs also supported increased bone regeneration in calvarial critical-size defects [5].

Adipose-derived stem cells (ADSCs) are multipotent with the ability to undergo multilineage differentiation and self-renewal. ADSCs offer several advantages over MSCs including ease of isolation, relative abundance, and rapidity of *in vitro* expansion [6]. Previous studies have demonstrated that ADSCs can play an important role in regenerative medicine, such as in applications to promote wound healing, increase vertical bone regeneration, and repair cartilage defects [7,8,9]. In addition, ADSCs have been shown to express a variety of paracrine factors that are known to be angiogenic and to have the ability to support angiogenesis *in vitro* through the secretion of vascular endothelial growth factor (VEGF)-A and VEGF-D [10]. Interestingly, ADSCs also can stimulate the generation of significantly more granulocytes and progenitor cells from human hematopoietic stem cells (HSCs) than MSCs *in vitro*, and ADSCs also can facilitate the homing of mouse HSCs to bone marrow better than MSCs *in vivo* [11]. Furthermore, ADSCs have been proven to have more potent proangiogenic activity than MSCs and to be more resistant to apoptosis *in vitro* [12,13]. Therefore, we hypothesized that ADSCs can support bone generation when applied within a tissue engineering strategy.

Cell sheets, which represent a relatively new method in tissue engineering, have been widely tested in attempts to regenerate various tissues, including skin, heart muscle, cartilage, and periodontium [14,15], because cells expanded in sheets are harvested together with their autocrine extracellular matrix (ECM) and intact cell–cell connections [14,16]. Moreover, MSC sheets have demonstrated beneficial effects in bone regeneration and reconstruction based on observations of high alkaline phosphatase activity and osteocalcin content [4,16], but techniques are needed to promote their osteogenic capacity.

Combining the osteogenic capacity of MSC sheets with the advantages of the proangiogenic potential of ADSCs, we developed a new strategy to fabricate vascularized bone grafts. We hypothesized that the addition of ADSCs to MSC sheets would support well-vascularized engineered bone formation without the need for an exogenous scaffold.

## 2. Results

### 2.1. Characterization of Adipose-Derived Stem Cells (ADSCs) and Mesenchymal Stem Cell (MSC) Sheets

**Figure 1:**
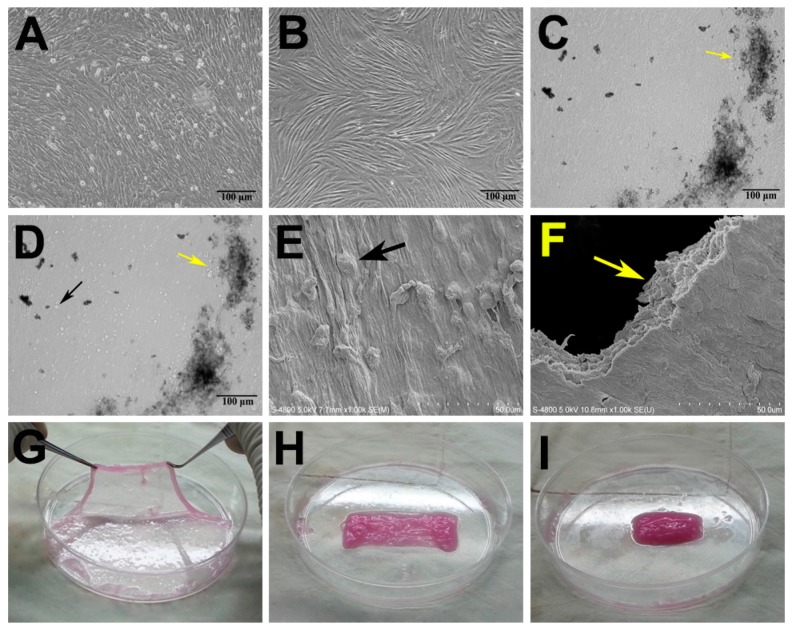
Characterization of adipose-derived stem cells (ADSCs) and mesenchymal stem cell (MSC) sheets. (**A**–**D**) Phase contrast microcopy. (**A**) MSCs presented a fibroblast-like morphology and closely spaced growth; (**B**) The fusiform ADSCs grew well with a spiral-shaped arrangement; (**C**) After MSCs were cultured in osteogenic medium for two weeks, they grew into multiple layers containing many calcium nodules (yellow arrow); (**D**) ADSCs adhered well to the MSC sheets upon seeding (black arrow); (**E**) Scanning electronic microscopy (SEM) examination demonstrated distinct mineral-like nodules on the surfaces of the cell sheets and the presence of endogenous extracellular matrix (ECM) in the cell sheets (black arrow); (**F**) Section analysis of SEM images of MSC sheets also indicated that they were composed of several layers of cells and their own ECM (yellow arrow); (**G**) MSC sheets could be easily lifted intact from the culture dish; (**H**) folded into a rectangle shape; and (**I**) rolled into a cylindrical construct.

Both MSCs and ADSCs in culture exhibited the typical characteristics of stem cells, including the ability to proliferate rapidly and self-renew. The fusiform ADSCs grew well in a spiral-shaped arrangement, whereas MSCs presented a fibroblast-like morphology and closely spaced growth (Figure 1A,B). After MSCs were cultured in osteogenic medium for two weeks, they grew into multiple layers and extension calcium nodule formation was observed (Figure 1C,D). ADSCs adhered well to the MSC sheets when they were seeded onto the sheets as shown in Figure 1D. Scanning electron microscopic examination demonstrated the presence of distinct mineral-like nodules on the surfaces of the MSC sheets (Figure 1E), and the MSCs were embedded in their own endogenous ECM (Figure 1E). Moreover, section analysis of MSC sheets confirmed that they were composed of several layers of cells and their ECM (Figure 1F). MSC sheets easily could be lifted intact from the culture dishes using a cell scraper and folded into a rectangle shape for subsequent rolling into cylindrical constructs that could be easily implanted (Figure 1G–I).

### 2.2. Gross Morphology and Micro Computed Tomography (CT) Analysis of Bone Formed in Vivo

**Figure 2:**
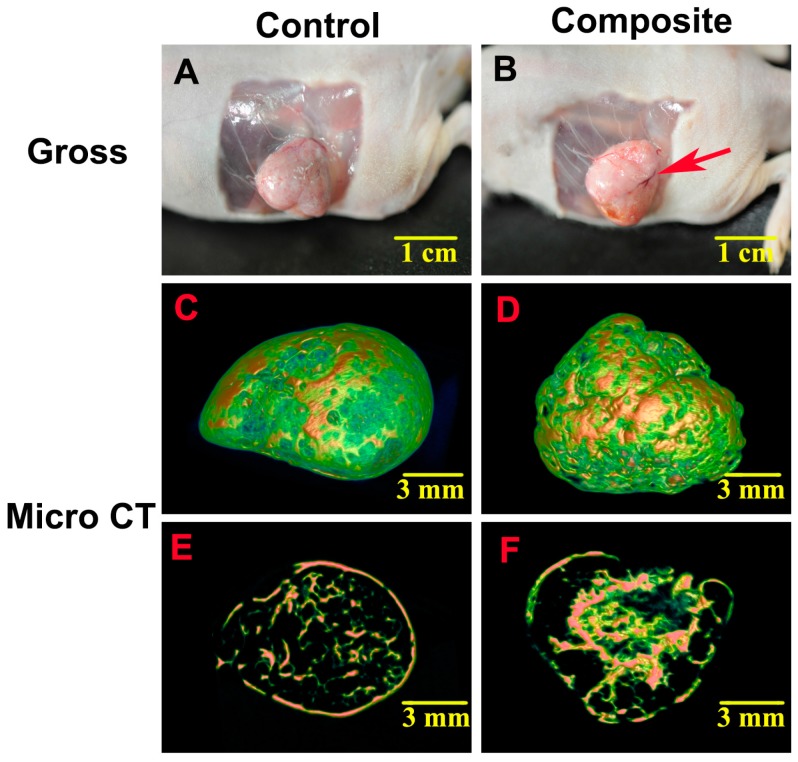
Gross morphology and micro computed tomography (CT) analysis of bone tissue formed *in vivo*. (**A**,**B**) On gross examination, the harvested specimens in the composite group were harder than those in the control group, and newly formed blood vessels were clearly observed in the tissue in the composite group (red arrow); (**C**,**D**) Micro CT images showed that the spongy bone structure in the composite group was much denser than that in the control group; (**E**,**F**) The cross-sectional micro CT images also indicated that the composite constructs generated more newly formed bone-like tissue than the MSC-only constructs.

At eight weeks after implantation, ectopic bone-like tissue had formed in both the control group and the composite group. The newly formed tissue in all specimens was intact, but the cylindrical shape had changed, which may have been due to the pressure of the surrounding skin at the transplant sites of the severe combined immunodeficiency (SCID) mice and the release and/or absorption of moisture in the constructs related to the culture medium. On gross examination, the harvested specimens in the composite group were harder than those in the control group, and newly formed blood vessels were clearly observed in the tissue in the composite group (Figure 2A,B).

**Figure 3:**
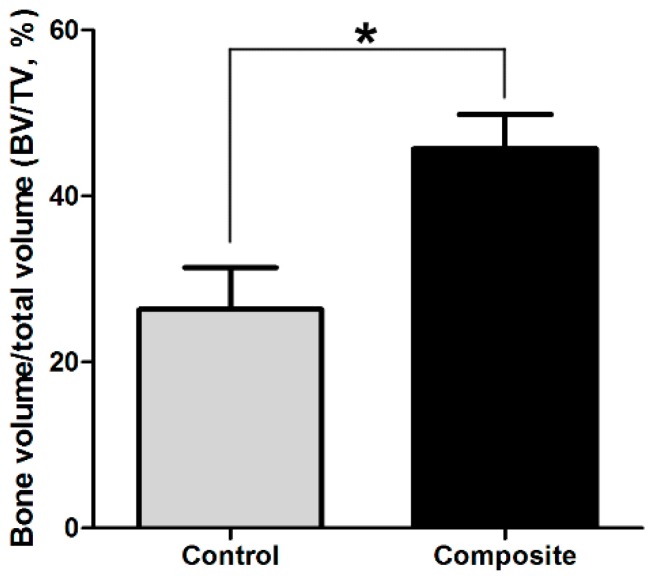
Comparisons of bone volume to total volume (*BV*/*TV*, %). The three-dimensional *BV*/*TV* values calculated based on the micro CT images differed significantly between the newly formed bone in the composite and control groups (* *p* < 0.05).

Micro CT images showed a mass of mineral deposition in both groups, but the spongy bone structure in the control group (Figure 2C) was much more sparse than that in the composite group (Figure 2D). Cross-sections of micro CT images also indicated that there was much more newly formed bone-like tissue in the composite group (Figure 2E,F). Additionally, the three-dimensional bone volume to total volume (*BV*/*TV*) values calculated from the micro CT images indicated significant differences in the amount of bone formed between the composite and control groups (*p* < 0.05; Figure 3).

### 2.3. Histological Examination of Newly Formed Bone Tissue

**Figure 4:**
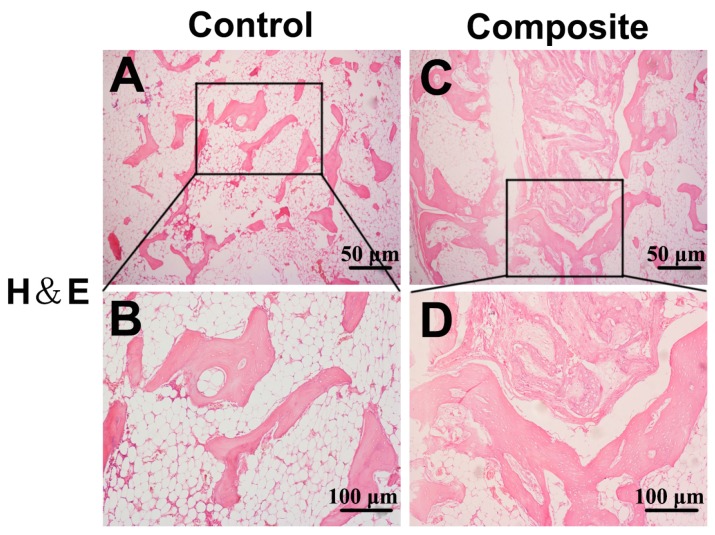

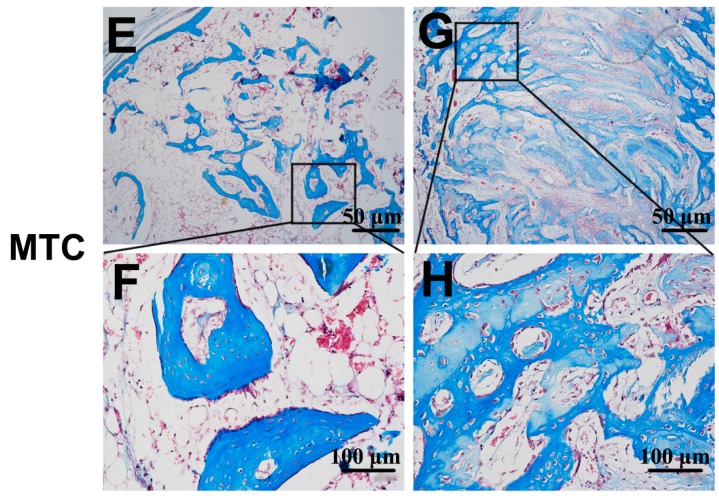
Histological examination of newly formed bone tissue. H&E staining: (**A**,**B**) In the control group, fewer newly formed island-like bone tissue structures were observed, and the neo-bone and cartilage-like tissues were sparse and unevenly distributed; (**C**,**D**) In contrast, in the composite constructs, a large number of interconnected ossified trabeculae and woven bone structures were observed in the peripheral regions of the constructs and large amounts of cartilage-like tissue were also observed in the central regions of the composite constructs. In addition, osteoblasts were observed in the neo-mineralized tissue and osteocytes were observed embedded in the dense matrix. On Masson’s trichrome (MTC) staining, the control group (**E**,**F**) also showed less osteogenesis than the composite group (**G**,**H**). (**A**,**B**,**E**,**F**) Low-magnification, 40×; (**C**,**D**,**G**,**H**) high-magnification, 200×.

**Figure 5:**
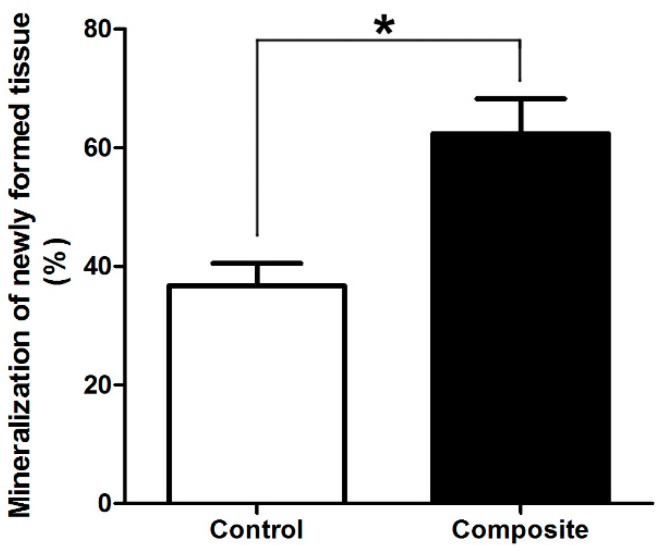
Comparisons of newly formed mineralized tissue. The percent area of new bone and cartilage in the control group was significantly lower than that in the composite group at eight weeks post-implantation (* *p* < 0.05).

Histologically, no obvious inflammation was observed in either group. Both osteogenesis and angiogenesis were observed in the control and composite groups, but obvious differences could be seen in the newly formed bone between the groups. The control group showed fewer newly formed island-like bone tissue structures, and the neo-bone and cartilage-like tissues were sparse and unevenly distributed (Figure 4A,B). In contrast, in the composite group, a large number of interconnected ossified trabeculae and woven bone structures were observed in the peripheral regions of the construct and large amounts of cartilage-like tissue were observed in the central regions (Figure 4C,D). In addition, osteoblasts were observed in the neo-mineralized tissue and osteocytes were embedded in the dense matrix. Histomorphometrical examination of the eight-week specimens demonstrated that mineralized bone and cartilage occupied 62.3% ± 6.8% and 36.7% ± 4.2% of the total cross-sectional areas of specimens of the composite and control groups, respectively (*p* < 0.05; Figure 5). The percent area of new bone and cartilage in the control group (Figure 4E,F) was significantly lower than that in the composite group (Figure 4G,H) at eight weeks post-implantation (*p* < 0.05).

## 3. Discussion

The results of the present study demonstrate that the addition of ADSCs to osteogenic MSC sheets led to improved bone formation *in vivo*, and thus, this novel method holds potential for bone tissue engineering. The major advantages of our method compared with the most common approaches previously reported for constructing tissue-engineered bone are as follows. First, after *in vitro* expansion the MSCs were harvested as an intact cell sheet using a cell scraper without the need for enzymatic digestion, which preserved the self-secreted ECM and cell-cell connections; Second, the ADSCs, which were applied as a source of angiogenic growth factors in this study, are easily accessible, abundant, and could be prepared from the same donor as the MSCs, which provides good compatibility; Third, our method did not require an exogenous scaffold as a cell carrier due to the inherent structure of our multi-cell constructs; Finally, MSC sheets including ADSCs could be formulated for injection, which would make the implantation procedure minimally invasive, resulting in a simpler surgery with less potential for scar formation.

Cell sheet technology already has been shown to be effective in tissue engineering applications [3,17]. For example, constructs engineered from cell sheets have been applied in the development of alternative therapies based on endoscopic transplantation, combinatorial tissue reconstruction, and polysurgery, in order to overcome shortcomings of cell delivery and regenerative therapies using conventional approaches [18]. In addition to MSC sheets, previous studies have investigated the abilities of sheets of human amniotic fluid stem cells and sheets of ADSCs to restore cardiac function and regenerate adipose tissue, respectively [19,20]. Moreover, MSC sheets without an exogenous scaffold have been used to construct engineered bone [16,21]. In our study, we confirmed that MSC sheets could form bone at an ectopic site through micro CT examination (Figure 2A,C,E) and histological analysis (Figure 4A,E). However, the amount of newly formed bone was small and not likely to satisfy the needs for bone defect repair, a finding that is in accordance with our previous study [22].

Based on the aforementioned advantages, ADSCs have been broadly applied in research to engineer tissues such as adipose, bone, and cartilage tissue, among others [6,23,24]. Moreover, ADSCs have been studied as a source of cells for the treatment of airway allergic diseases, autoimmune diseases, and chronic stroke based on their potential to stimulate neovascularization [25,26]. In the present study, the osteogenic potential of composite constructs containing ADSCs on MSC sheets upon subcutaneous implantation in SCID mice was significantly greater than that of MSC sheets without ADSCs, as evidenced by the results of our micro computed tomography (CT) analysis (Figure 2) and histological examination (Figure 4). The reasons for these effects may be that the seeded ADSCs increased the amount of stem cells in the sheets, and ADSCs differentiated into osteoblasts and osteocytes [9]. Second, ADSCs are known to secrete many paracrine factors that stimulate angiogenesis, including VEGF-A, VEGF-D, and others [8,10,11,14]. Third, ADSCs have superior tolerance to oxidative stress and a better capacity to resist apoptosis than MSCs, and thus, ADSCs may be able to prevent MSC apoptosis to some extent [7]. Finally, a synergistic effect may exist when MSCs and ADSCs are used together, which maintains the properties of stem cells and increases their osteogenic and angiogenic potential [27].

Although our results confirm that ADSCs can significantly increase the osteogenic potential of MSC sheets at an ectopic site within eight weeks after implantation, our study still has several limitations. For example, experiments testing the ability of our composite constructs to repair bone defects in an animal model should be performed, because our present study only tested the effects of ADSCs on osteogenic MSC sheets in SCID mice. Furthermore, the molecular mechanisms responsible for the increase in the osteogenic potential of MSC sheets and ADSC complexes should be studied in the future.

## 4. Experimental Section

### 4.1. Ethical Approval

This study was reviewed and approved by the Institutional Animal Care and Use Committee (the project identification code was 2015 (kq-034), which was approved in 06-03-2015) of School of Stomatology at the Fourth Military Medical University (FMMU), Xi’an, China.

### 4.2. MSC Isolation, Culture, and Sheet Preparation

New Zealand rabbits (three months old, with an average weight of 2.5 kg) were provided by the animal holding unit of FMMU. All efforts were made to minimize their suffering, and they were taken care of in accordance with established institutional guidelines. MSCs were harvested and isolated from the ilium marrow of rabbits as reported previously [28,29]. Briefly, ilium marrow (approximately 3 mL) was suspended in low-glucose Dulbecco’s modified Eagle’s medium (DMEM; Gibco, Carlsbad, CA, USA) supplemented with 10% fetal bovine serum (FBS) (Gibco), 2% antibiotics (200 mg/mL penicillin and 200 mg/mL streptomycin; Gibco), and L-glutamine (0.272 g/L, Sigma-Aldrich, St. Louis, MO, USA). The cells were seeded in 100-mm cell culture dishes, and standard DMEM was added to reach a volume of 15 mL for culture of the cells at 37 °C with 5% CO_2_ and 100% humidity. Non-adherent cells were removed during the first medium change 3 days later. After that, the medium was changed every 3 days until the adherent cells reached greater than 80% confluence. Then the cells were digested with 0.25% trypsin (Hyclone, Logan, UT, USA) and sub-cultured at a ratio of 1:3. Passage two cells were used for subsequent experiments.

Second passage MSCs were seeded into 100-mm dishes at a density of 5 × 10^4^ cells/cm^2^ in osteogenic medium composed of standard DMEM containing 50 mg/mL l-ascorbic acid 2-phosphate, 100 nM dexamethasone, and 10 mM β-glycerophosphate (Sigma-Aldrich). The cells were incubated at 37 °C with 5% CO_2_ and cultured continuously for 14 days without passaging. During this continuous culture period, the osteogenic medium was changed every 2 days. After 14 days in culture, MSC sheets were formed, and they could be easily isolated from the dishes with a cell scraper. For characterization of MSC sheets, small pieces of cell sheets were processed for observation by scanning electronic microscopy (SEM) (S-4800, Hitachi, Japan).

### 4.3. ADSC Isolation and Culture

ADSCs were harvested and isolated from the inguinal adipose tissue pouch of the same rabbits employed for MSC isolation as reported previously [28,30]. First, phosphate-buffered saline (PBS) was used to wash the obtained adipose tissue twice to remove remnant blood. Secondly, the adipose tissue was carefully shredded using sterilized surgical scissors, and then the minced adipose tissue was subjected to digestion with 2 mg/mL collagenase type I (Sigma-Aldrich) in PBS at 37 °C under continuous shaking. After 1 h of digestion, high-glucose DMEM supplemented with 10% FBS, 2% antibiotics (200 mg/mL penicillin and 200 mg/mL streptomycin), and l-glutamine (0.272 g/L) was added to neutralize enzymatic activity. Then the tissue debris was removed by filtering through a 200-μm mesh filter. Lastly, the digested cells were pelleted via centrifugation for 5 min at 1000 rpm, resuspended in high-glucose DMEM with 10% FBS, seeded into 25-cm^2^ flasks at a density of 2.5 × 10^4^ cells/mL, and incubated at 37 °C with 5% CO_2_ and 100% humidity. The floating cells were also removed, and the medium was changed every 2 days. The cells were digested with 0.25% trypsin (Hyclone) when they reached greater than 80% confluence and subcultured at a ratio of 1:3. Cells of the third passage were used in subsequent experiments.

### 4.4. Preparation of MSC Sheet–ADSC Constructs

ADSCs (1 × 10^6^) suspended in 500 μL medium were seeded onto osteogenic MSC sheets, and the cells were co-cultured for 4 h to ensure adherence of ADSCs to the sheets. Constructs were prepared by folding individual sheets containing ADSCs and rolling them into cylindrical constructs. Eight constructs were prepared from MSC sheets containing ADSCs, and eight constructs were prepared from MSC sheets not containing ADSCs for use as control constructs.

### 4.5. Surgical Protocol

Severe combined immunodeficiency (SCID) mice originally obtained from the animal center of the Fourth Military Medical University were bred and maintained in pathogen-free conditions. All procedures for handling mice were reviewed and approved by the Animal Care Committee of the Fourth Military Medical University. A total of 16 transplantation sites on eight male, 6-week-old SCID mice, weighing 20–25 g, were randomly assigned to two groups (*n* = 8 in each group): the control group (MSC sheets without ADSCs) and the composite group (MSC sheets with ADSCs). Shortly after the mice were anesthetized with 3% isoflurane gas, the control and composite constructs were implanted into the subcutaneous sites on the backs of the mice.

### 4.6. Micro Computed Tomography (CT) Analysis

At 8 weeks after transplantation, all mice were euthanized with an overdose of anesthesia. Micro-CT system (Siemens Inveon Micro CT, Siemens AG, Munich, Germany) imaging was performed on the backs of SCID mice for the purpose of evaluating changes in the tissue constructs before they were harvested for histological analysis. Samples were scanned with a resolution of 0.22 mm, and 692 scan slices were obtained and reconstructed on the basis of the manufacturer’s references. The output was represented as three-dimensional stacks using Inveon Research Workplace (Siemens AG). The thresholds employed in our study were 68–1732 Hounsfield Units (HU) for cortical bone and −70–67 HU for cancellous bone, grounded on threshold measurements for examples of porcine femur bone [31]. Bone volume/total volume (*BV*/*TV*) ratios also were measured.

### 4.7. Histological Analysis of Implanted Constructs

After micro CT scanning, the newly formed constructs were harvested and examined macroscopically. The samples were then fixed in 4% paraformaldehyde for 24 h and divided into two parts. One half of each sample was decalcified for 2 weeks in 10% EDTA (ethylenediaminetetraacetic acid; pH 8.0) prior to being embedded in paraffin, sectioned at 4 μm thickness, stained with hematoxylin and eosin (H&E), and observed by light microscopy (DX51, Olympus, Tokyo, Japan).

The other half of each sample was fixed in 10% formaldehyde for 1 week at room temperature. The specimens were dehydrated in a graded ascending series of ethanol (70%–100%) and then saturated and embedded in methyl methacrylate. A high-speed precision microtome (Leica SP1600, Wetzlar, Germany) was used to prepare serial sections (150 μm). All sections were ground to a thickness of 35 μm, stained with Masson’s trichrome, and observed by light microscopy.

Sections were selected from each sample for histomorphometrical examination as reported previously [29]. First, Masson’s trichrome staining was the base of this examination. A light microscope (Leica Microsystems AG) was utilized for observation. Two unbiased observers who were blinded to the experimental factors randomly observed and recorded three high-resolution, low-magnification images from each section and then examined these images twice by using computer-based image analysis techniques (Leicas Qwin Pro-image analysis system, Wetzlar, Germany). The cross-sectional areas of newly formed mineralized bone and cartilage (blue staining) were calculated and represented as relative percentages of the entire cross-sectional areas.

### 4.8. Statistical Analysis

All quantitative data are expressed as mean ± standard deviation (SD) values. Statistical analysis was conducted using the GraphPad Prism 5 package (GraphPad Software, Inc., La Jolla, CA, USA), and analysis of variance (ANOVA) was used for statistical analysis. *p* values less than 0.05 indicated that differences were statistically significant.

## 5. Conclusions

We have developed a novel method based on the combination of osteogenic MSC sheets and ADSCs for generating tissue-engineered bone. ADSCs, due to their distinctive advantages, significantly increased the osteogenic potential of MSC sheets implanted at an ectopic site in SCID mice. Although further research is needed, the current study indicates that our novel constructs are a promising therapeutic strategy to improving bone regeneration.
